# Behavioral assessment of blue-fronted Amazon parrots (*Amazona aestiva* Linnaeus, 1758) subjected to experimentally induced osteoarthritis and rescue analgesia with tramadol in a laboratory environment

**DOI:** 10.3389/fvets.2026.1802230

**Published:** 2026-05-14

**Authors:** Isaac Mourao Xavier, Cristiano Schetini de Azevedo, Cristiane Schilbach Pizzutto, Bruna Tizoni Guedine, Sônia Regina Pinheiro, Denise Tabacchi Fantoni

**Affiliations:** 1 Department of Surgery, Faculty of Veterinary Medicine and Animal Science, University of São Paulo, São Paulo, Brazil; 2 Departamento de Evolução, Biodiversidade e Meio Ambiente, Instituto de Ciências Exatas e Biológicas, Universidade Federal de Ouro Preto, Campus Morro do Cruzeiro, Ouro Preto, Brazil; 3 Laboratório de Ecologia e Evolução, Instituto Butantan, Butantã, São Paulo, Brazil; 4 Programa de Pós-Graduação em Reprodução Animal, Faculdade de Medicina Veterinária, Universidade de São Paulo, Cidade Universitária Armando Salles de Oliveira, São Paulo, Brazil; 5 Faculty of Veterinary Medicine and Animal Science, University of São Paulo, São Paulo, Brazil

**Keywords:** avian model, joint inflammation, pain management, psittacidae, welfare

## Abstract

**Introduction:**

This study aimed to identify behavioral changes in three blue-fronted Amazon parrots (*Amazona aestiva*) subjected to experimental induction of osteoarthritis and subsequent tramadol rescue analgesia.

**Methods:**

All parrots received injections of 0.1 mL of 8% monosodium urate monohydrate (MUM) solution into the left intertarsal joint. Behaviors were recorded during all study phases (1: baseline; 2: post-induction of osteoarthritis; 3: post-induction of osteoarthritis with tramadol 1; 4: post-induction of osteoarthritis with tramadol 2) using focal sampling with instantaneous recordings every 30 s during 20-min sessions. Data were analyzed using generalized linear mixed models.

**Results:**

Observed changes in behavioral patterns, including decreased locomotion, and exploration, increased stopped and inactive behavior, and reduced cage use (fewer areas used during acute pain), followed by partial recovery after tramadol administration, were consistent with the manifestation of unilateral arthritic pain and its possible attenuation.

**Discussion:**

The species-specific ethogram for arthritic pain developed in this study may contribute to future antialgic evaluations with tramadol and other drugs, supporting the future development of a pain assessment scale for blue-fronted Amazon parrots.

## 1 Introduction

The International Association for the Study of Pain (IASP) has defined pain as an unpleasant sensory and emotional experience associated with actual or potential tissue injury (1). Animals modify their behavior and physiology to minimize pain and injury, prevent recurrence, and facilitate recovery (2). However, it is not always easy for humans to recognize pain. In birds, the absence of facial expressions and a verbal language shared with humans makes it difficult to translate the perception of pain (3). In addition, birds tend to disguise pain as a defense mechanism against predators (4, 5).

Recognizing and assessing the behavioral changes associated with the pain process in birds is essential for effective pain management in laboratory, clinical, and zoological environments, as well as in the context of pet care (6–8). If pain is not measured accurately, it may be underestimated, and the therapy adopted may prove to be insufficient (9, 10). Consequently, an under-treated patient would experience negative repercussions, such as increased morbidity, mortality and compromised quality of life, which also impacts the life of their caregiver (11).

Although more complex than physiological parameters (heart rate, respiratory rate, blood pressure, leukograms, electroencephalograms, and fecal and plasma corticosterone), behavioral indicators of pain have the advantage of being externally detectable and shorter, since they do not require as much time or processing to quantify (6, 12). Because of this, recognizing the behaviors associated with pain facilitates its assessment, quantification, and treatment. Behavioral responses can exhibit consistent patterns across species, with some individual variation. Regardless of the similarity of the painful stimulus, such manifestations can be extrapolated only with difficulty across species (6, 8, 13).

In the early 2000s, the scarcity of valid methods for analyzing pain in farm, companion, laboratory and wildlife animals was the subject of an international workshop (8). In the years that followed, little progress was made in the field of avian species; some studies have emerged on Hispaniolan parrots (*Amazona ventralis*), green-cheeked conures (*Pyrrhura molinae*), chickens (*Gallus gallus domesticus*), domestic pigeons (*Columba livia*), and wild ducks (*Cairina moschata domestica*) in which it was proposed to use osteoarthritis induced under laboratory conditions as a model (7) to help assess pain and treatment in these species. These studies found that among the behaviors associated with experimentally induced arthritic pain in Psittaciformes (*A. ventralis* and *P. molinae*) is a decrease in locomotion, perching, and the ability to bear weight on the affected pelvic limb. Increased feather ruffling, cleaning of the arthritic limb and attempts to reach snacks offered in the cage have also been reported (14, 15).

Classified as Near Threatened due to significant illegal trafficking, the blue-fronted Amazon parrot (*Amazona aestiva*) is nonetheless one of the most commonly found parrot species as pets in Brazil (16, 17). However, despite being quite common, studies on the pain-indicating behaviors of this species are nonexistent. Joint uric gout, for example, is reported to be prevalent in senile psittacine and is known to be a process that causes chronic pain (18). The treatment of psittacine birds diagnosed with this clinical condition, including the blue-fronted Amazon parrot, has been based on extrapolating the data obtained from models of induced osteoarthritis in Hispaniolan parrots and green-cheeked conures. However, as previously noted, the different species exhibit distinct characteristics in terms of what is considered normal behavior, pain manifestations, and responses to treatment, including drugs, doses, and frequencies. Given this, it is essential to implement this experimental model (osteoarthritis induced under laboratory conditions) in other species to provide a basis for understanding pain and its treatment.

The antinociceptive action of tramadol has been demonstrated in different avian species, as well as its usefulness in controlling induced pain (19, 20). It is an atypical opioid whose analgesic action derives from the inhibition of serotonin and noradrenaline reuptake, as well as the agonism of its primary metabolite, O-desmethyltramadol, at the μ receptor (21, 22). This drug is recommended for the management of mild to moderate pain and can be administered to birds orally, intramuscularly, and intravenously (20). At a dose of 30 mg/kg administered orally, tramadol proved effective in treating pain resulting from osteoarthritis induced in wild ducks, promoting effective antinociception for up to 6 h in Hispaniolan parrots (23, 24).

Based on these findings, the application of an experimental model of induced osteoarthritis in blue-fronted Amazon parrots can contribute and add vital information to the study of the efficacy and efficiency of drugs intended to treat pain in this avian species. Thus, this study aimed to identify behavioral changes in three blue-fronted Amazon parrots subjected to experimental induction of osteoarthritis and subsequent tramadol rescue analgesia. We hypothesize that the parrots will become less active and use the cage less during the induced pain phase, and that the display levels of most behaviors will return to baseline levels due to the tramadol treatment, which will alleviate the birds' pain. This result will be reinforced by increased cage use in the phases with tramadol administration.

## 2 Material and methods

### 2.1 Ethical statement

The research was authorized by the Chico Mendes Institute for Biodiversity Conservation (ICMBIO) (No. 79318-3) and the Ethics Committee for the Use of Animals (CEUA) (No. 1417010421) of the Faculty of Veterinary Medicine and Zootechny (FMVZ) of the University of São Paulo (USP). The study was registered with the National System for the Management of Genetic Heritage and Associated Traditional Knowledge (SISGEN) (No. AC6CB00).

### 2.2 Study place, animals, housing, and maintenance

The study was conducted in one laboratory of the Surgery Department of USP's FMVZ, located in the Municipality of São Paulo, southeastern Brazil (-23°3′36.7“ S; 46°3′58.7” W). Three adult specimens of the blue-fronted Amazon parrot (parrots 1, 2 and 3) from a commercial aviary were used. Birds were acclimatized for 21 days in the laboratory. The criteria adopted for inclusion of the individuals were: a body condition score of three or four, equivalent to the pectoral musculature at the level of the keel and the same musculature with a slight projection in relation to the carina, respectively (25), the adult stage of development, the absence of clinical signs suggestive of systemic disease, the normality of copro-parasitological tests, complete blood count, liver biochemistry (Aspartate Aminotransferase - AST, Creatine Kinase - CK, Lactate Dehydrogenase - LDH and biliary acids) and kidney biochemistry (uric acid), and the integrity of the locomotor system on radiographic examination.

**Figure 1:**
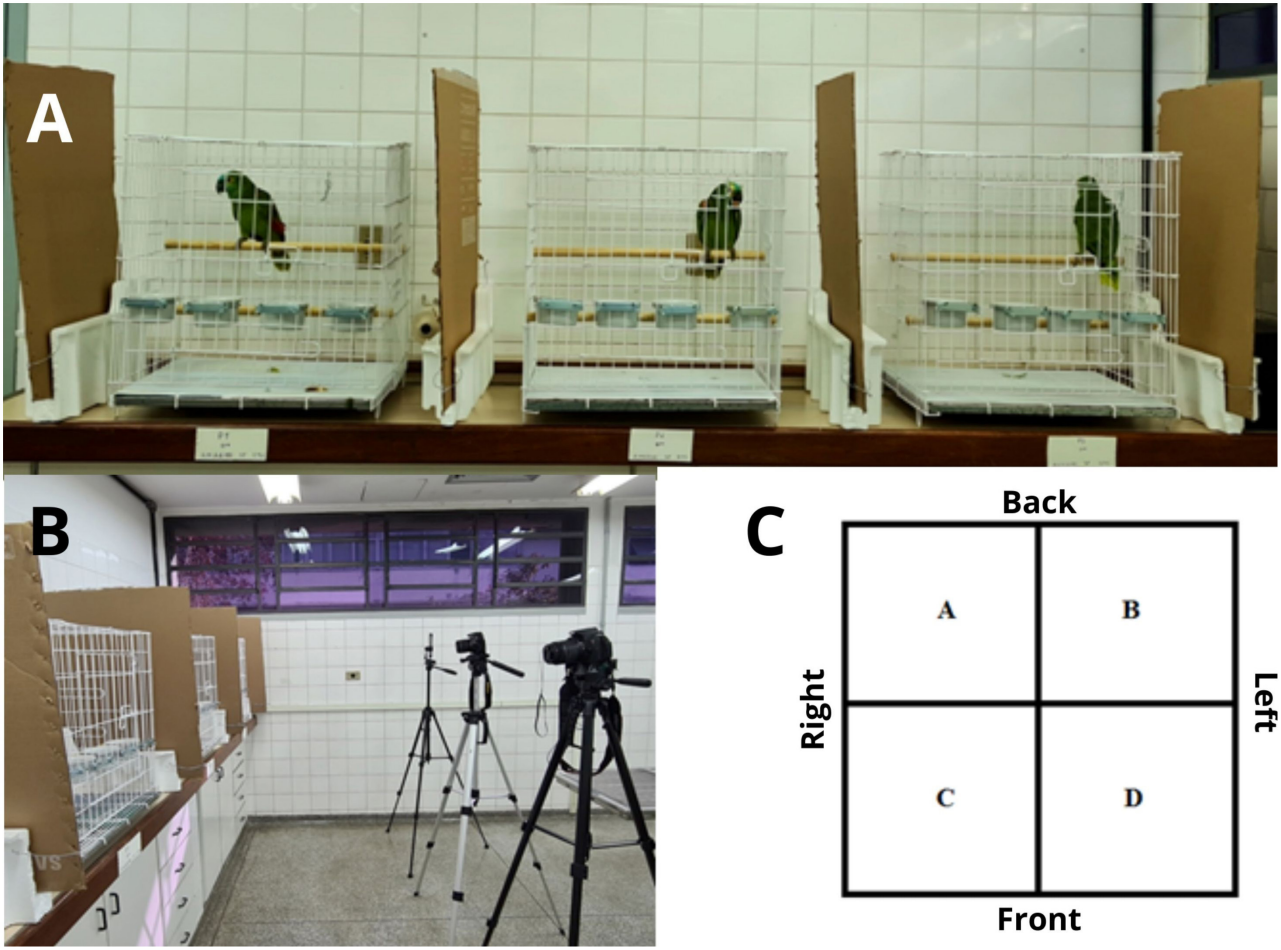
**(A)** Cages used to collect behavioral data from the blue-fronted Amazon parrots during the pain induction and tramadol control experiments, showing the layout of the cages and the cardboard sheets that were used to prevent visual contact between the birds. **(B)** Video recording devices attached to tripods and directed at the cages. **(C)** Schematic drawing of the top view of the cages, showing virtual quadrants A, B, C and D.

Each bird was housed in a 1 m^3^ metal cage with two 2.5 cm diameter wooden perches positioned separately. The lower perch, located at the front of the cage, was 35 cm high, while the upper perch, placed at the back of the cage, was 70 cm from the floor. In addition, in front of the lower perch, a drinking fountain, a feeder for extruded feed, a feeder for snacks, and a feeder for fruit were attached, from right to left, on the front grid. They were all circular and made of porcelain. Water, fruit (papaya and banana) and extruded parrot food (BIOTRON^^®^^) were provided *ad libitum*. Feeding and sanitation took place between 6:00 a.m. and 8:00 a.m. and between 4:00 p.m. and 6:00 p.m. A photoperiod of 12 h:12 h light:dark was established. A cardboard sheet was placed 25 cm from each side of the cages to ensure the visual isolation of the individuals (Figure 1A). Three video recording devices were positioned: an EOS Rebel T5i (CANON^^®^^), a D5500 (NIKON^^®^^) and a BISON X10 Pro (UMIDIG^^®^^), attached to tripods so that each was 1.5 m from the front of the cage (Figure 1B). Additionally, each cage was virtually divided into four quadrants (Figure 1C). The use of specific structures and areas in the cage was also observed, including the anterior grid (Ant grid), drink fountain (Drink fount), fruit feeder (Fruit), inferior grid (Inf grid), inferior perch (Inf perch), left anterior corner (Left ant corner), left grid, left posterior corner (Left post corner), posterior grid (Post grid), ratio feeder (Ratio), right anterior corner (Right ant corner), right grid, right posterior corner (Right post corner), superior grid (Sup grid), superior perch (Sup perch), and treat feeder (Treat).

### 2.3 Preparation of the monosodium urate monohydrate (MUM) solution

In partnership with the Pharmacy Department of USP's Faculty of Pharmaceutical Sciences (FCF), an 8% solution of MUM crystals was prepared, following the same protocol as published studies on the experimental model of osteoarthritis in Hispaniolan parrots and green-cheeked conures (14, 15, 26). Following this, 3 mg of the final solution was subjected to thermogravimetric analysis (TGA) using the DSC-7020 High Sensitivity Differential Scanning Calorimeter (EXSTAR^®^) to characterize the material obtained by thermal treatment (27). Finally, to confirm the solution concentration based on the absorbance of the MUM crystals (28), a 20 mg aliquot was analyzed by ultraviolet-visible (UV-Vis) spectroscopy using an Evolution 201 UV-Vis Spectrophotometer (THERMO SCIENTIFIC^®^). The tests indicated that the 8% MUM solution was successfully produced.

### 2.4 Intra-articular injection training in cadavers

Replacing the MUM solution with 1% methylene blue dye (PERFYL TECH^®^) in a 1 mL syringe (SR^®^) coupled to a 23 G needle (SR^®^), a volume of 0.1 mL was injected into both intertarsal joints of nine corpses of blue-fronted Amazon parrots. The same steps were followed as described in the protocol for inducing osteoarthritis in other parrot species using an 8% MUM solution (14, 15). Finally, each joint was dissected to verify whether correct access to the intertarsal joint cavity had been achieved, as confirmed by dyeing it blue, and whether the infused volume remained within it, which was indeed the case. The training was successful (Supplementary material 1).

### 2.5 Experimental design

The study was divided into four phases: (1) Baseline, (2) Post-induction of osteoarthritis, (3) Post-induction of osteoarthritis with tramadol 1, and (4) Post-induction of osteoarthritis with tramadol 2. Data collection occurred from 06:00 to 08:00, 09:00 to 09:30, and 17:15 to 17:45, as these were the periods of greatest activity for the parrots, based on preliminary observations.

#### 2.5.1 Phase 1: baseline

Before inducing osteoarthritis, parrot behaviors were evaluated over 11 days to obtain baseline data. To this end, the focal sampling and instantaneous recording methods were employed (29, 30). On a field sheet, the behavioral manifestations observed in videos obtained from 20-min recording sessions were recorded at a 30-s sampling interval.

#### 2.5.2 Induction of osteoarthritis

After the behavioral recordings of the baseline period, the parrots were physically restrained and transported individually to the surgical center of the Veterinary Hospital of USP's FMVZ. There, the 8% MUM solution was injected into one of the intertarsal joints of each bird to induce temporary, self-limiting osteoarthritis, simulating joint uric gout, which is common in senile psittacine birds. (14, 15, 18). This procedure was always conducted by the same trained researcher.

Initially, anesthesia was induced using 5% isoflurane (ISOFORINE^®^) in 1.5 L of oxygen per minute via a mask connected to the Fabius Tiro anesthesia machine (DRÄGER^®^). When the bird demonstrated adequate muscle relaxation, it was placed in the dorsal decubitus position. Once confirmed in the second plane of anesthesia (absence of interdigital reflex and relaxation of the gnathotheca), intubation was performed using a 2 mm endotracheal tube without a balloon (MILA^®^). Anesthesia was maintained with 2–3% isoflurane in 1.5 L of oxygen per minute (14). An inspired oxygen fraction of 100% was used.

In the distal region of the tibiotarsus joint and proximal tarsometatarsal joint of the left pelvic limb, which was not wearing the identification ring, the feathers were removed, and asepsis was carried out with 2% chlorhexidine (VIC PHARMA^®^). Using a 23 G needle (SR^®^), 0.1 mL of 8% MUM solution was injected into the intertarsal joint (Figure 1). The joint was then flexed and extended for 15–30 s (14). Finally, the inhalation anesthesia was stopped, extubation was performed, and, as soon as the straightening reflex was observed, the bird was placed back in its cage.

#### 2.5.3 Phase 2: post-induction of osteoarthritis

Following the induction of osteoarthritis, the behavior exhibited by the birds without the interference of rescue analgesia was recorded. To this end, the same baseline recording method was employed for 1 day (29, 30). The recording sessions were conducted in the first and second hours after induction, at 9.30 am and 10.30 am, and were repeated every 2 h until 4.30 pm for 1 day.

#### 2.5.4 Phase 3: post-induction of osteoarthritis with tramadol 1

Once arthritic pain had been established and reluctance to move and feed for more than an hour had been observed (14), rescue analgesia (tramadol - 7 mg/kg - IM) was instituted in the three individuals at 5.30 pm (9 h post-induction). The behaviors exhibited during the first hour after tramadol administration at 6:30 pm were recorded using the same protocol as in the previous phases (1 day).

##### 2.5.4.1 Phase 4: post-induction of osteoarthritis with tramadol 2

Twelve hours after the initial application of tramadol (21 h post-induction), tramadol was administered again, alternating the side of the pectoral muscles of the three parrots and using the same dose and route of administration. The recording sessions were resumed at 6:30 am, 1 h after tramadol administration, and continued every 2 h for 8 days. Like the previous stages, the focal sampling and instantaneous recording methods (every 30 s) were adopted (29, 30).

### 2.6 Ethogram

An ethogram containing eight behavioral categories was used: active standing, inactive standing, locomotion, maintenance, feeding, vocalization, exploration, and unwanted behaviors (Table 1). In each behavioral recording, information was also collected about the cage use, including the quadrant where the bird was located and the cage structures and areas used by the bird. The ethogram was constructed from three days of preliminary observations (30).

**Table 1:** Ethogram of the Blue-fronted Amazon parrots (*Amazona aestiva*) used to evaluate the effects of the experimental induction of osteoarthritis and rescue analgesia with tramadol in a laboratory environment.

Behavioral category	Description
Stopped active	The bird remained still, with its wings retracted, but continued to observe its surroundings, its head turned forward or to the side.
Stopped inactive	The bird remained without moving, resting (head rotated 90° to one side or facing forward, eyelids half closed, wings retracted, and feathers erect) or sleeping (head turned back and resting on its back, eyelids closed, wings retracted, and feathers erect).
Locomotion	The bird moved around the cage, vertically and/or horizontally.
Maintenance	The bird exhibited self-care behaviors, including preening of its feathers and beak, as well as behaviors related to bathing, scratching, stretching, flapping, yawning, and excreting.
Feeding	The bird consumed food items from the feeders.
Vocalization	The bird emitted vocal sounds.
Exploration	Interaction of the bird with elements of the cage, which could be manipulated, pecked, peeled, torn apart, or shredded. Parrot could use its tongue and/or foot.
Unwanted behaviors	The bird exhibited stereotypies, including pacing, circling, and performing vertical, horizontal, diagonal, and circular swings, as well as raising and lowering its head.

### 2.7 Data analysis

The data were compiled in spreadsheets and analyzed using descriptive statistics. Generalized Linear Mixed Models (GLMMs) were constructed, with the recorded behaviors being the response factors, the phases (baseline, post-induction of osteoarthritis, post-induction of osteoarthritis with tramadol 1, and post-induction of osteoarthritis with tramadol 2), the individuals (parrots 1, 2 and 3), and the interaction between these two variables as explanatory factors, and the day as a random factor. All models employed a negative binomial distribution of errors (using the glmmTMB package) (31). The “performance” package (32) was used to build the GLMMs. Tukey's *post-hoc* test, from the ‘emmeans' package (33). Likelihood Ratio Tests (LRTs) were used to assess whether the random variable ‘day' influenced the results observed in the GLMMs (using the lmtest package) (34). Figures were built using the ggplot2 package (35). All analyses were conducted using R 3.4.2 software (36), with a significance level of 95%.

Generalized Linear Mixed Models (GLMMs) were also constructed to evaluate the cage use by the parrots, with the cage quadrants being the response factors, the phases (baseline, post-induction of osteoarthritis, post-induction of osteoarthritis with tramadol 1, and post-induction of osteoarthritis with tramadol 2), the individuals (parrots 1, 2 and 3), and the interaction between these two variables as explanatory factors, and the day as a random factor. All analyses were conducted using the same packages described for the first GLMMs.

A Principal Component Analysis (PCA) was conducted to evaluate how the parrots behaved in each quadrant of the cage during each phase of the study. To do this, the ggplot2 (35), FactoMineR (37), and factoextra (38) packages were used. Generalized Linear Mixed Models (GLMMs) were also constructed to evaluate use of specific structures and areas in the cage by the parrots (the anterior grid, drink fountain, fruit feeder, inferior grid, inferior perch, left anterior corner, left grid, left posterior corner, posterior grid, ratio feeder, right anterior corner, right grid, right posterior corner, superior grid, superior perch, and treat feeder), with the cage specific structures and areas being the response factors, the phases (baseline, post-induction of osteoarthritis, post-induction of osteoarthritis with tramadol 1, and post-induction of osteoarthritis with tramadol 2), the individuals (parrots 1, 2 and 3), and the interaction between these two variables as explanatory factors, and the day as a random factor. All analyses were conducted using the same packages previously described for the GLMMs.

## 3 Results

The 122.94 h of video recordings of the three birds (60.99 h baseline, 4.98 h post-induction of osteoarthritis, 0.99 h post-induction of osteoarthritis with tramadol 1, and 55.98 h post-induction of osteoarthritis with tramadol 2) resulted in 14,752 behavioral recordings.

Overall, parrots displayed the most behaviors in the stopped active (mean ± SE: 16.57 ± 0.59) and maintenance (mean ± SE: 4.79 ± 0.27) categories, which contrasts with the low level of vocalizations (mean ± SE: 0.18 ± 0.04) and unwanted behaviors (mean ± SE: 1.84 ± 0.19). Stopped inactive (mean ± SE: 2.73 ± 0.31), locomotion (mean ± SE: 2.77 ± 0.16), feeding (mean ± SE: 3.64 ± 0.20), and exploration (mean ± SE: 2.25 ± 0.30) were exhibited at an intermediate level by the parrots.

**Figure 2:**
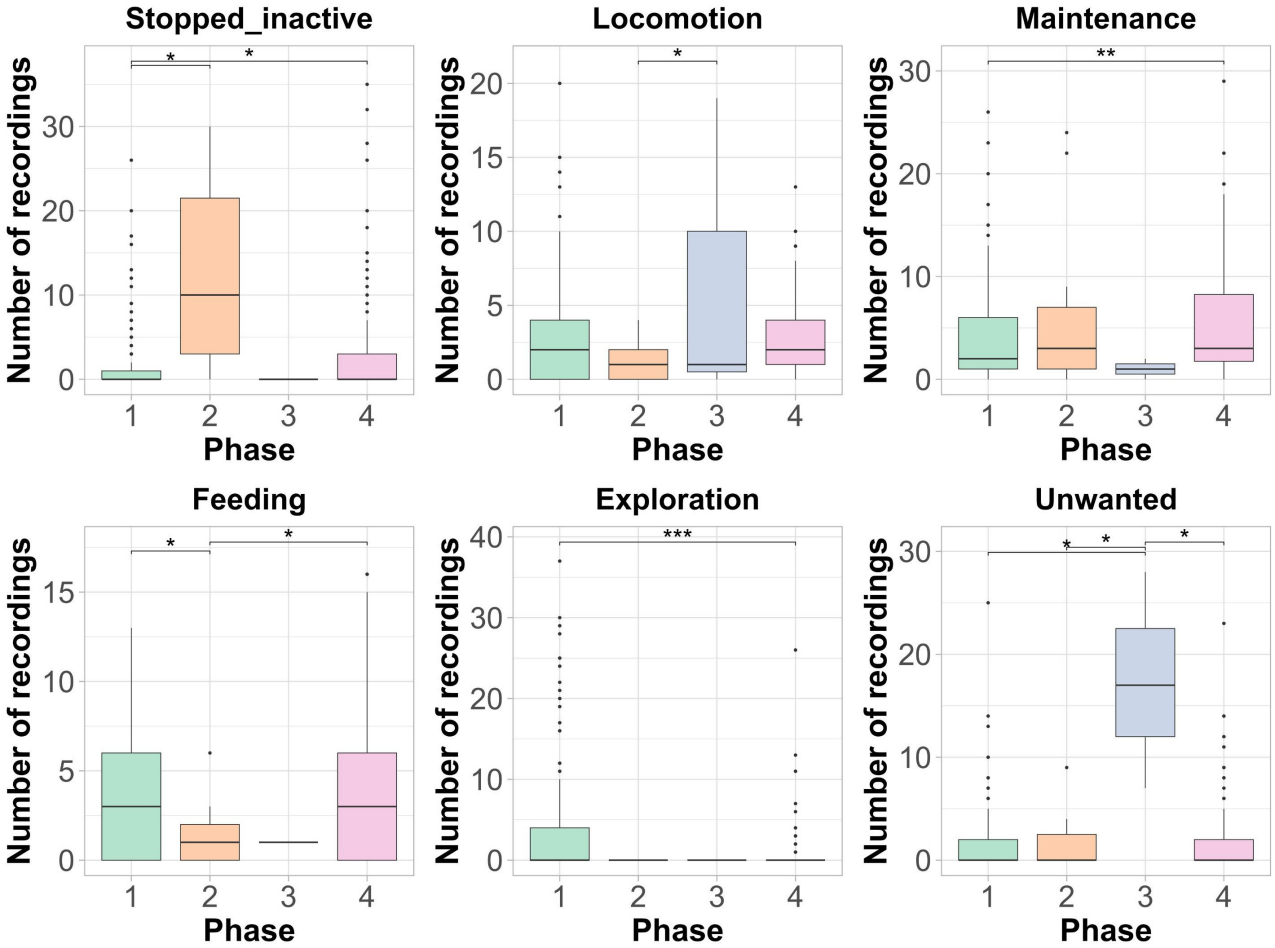
Results of the GLMM for the behaviors expressed by blue-fronted Amazon parrots (*Amazona aestiva*) during the experiment phases (1: Baseline, 2: Induction of osteoarthritis, 3: Post-induction of osteoarthritis with tramadol 1, 4: Post-induction of osteoarthritis with tramadol 2). * = *p* < 0.05; ** = *p* < 0.01; *** *p* < 0.001. Boxplots: The dark line in the center of the box represents the median, while the lower and upper edges of the box represent the first and third quartiles. The whiskers depict the range of the data, excluding outliers (shown as black dots).

There was no influence of the study phases on the expression of the categories, stopped active and vocalization. The study's phases influenced all other behaviors. Stopped inactive was more exhibited during phase 2 (induction of osteoarthritis), then in phases 1 (baseline) and 4 (post-induction of osteoarthritis with tramadol 2) (Figure 2). Locomotion and unwanted behaviors were exhibited most frequently during phase 4, compared to phases 2 and 1, respectively (Figure 2). Parrots consumed more food (feeding) during phases 1 and 4 compared to phase 2, while exploration was more evident during phase 1 compared to phase 4 (Figure 2).

**Figure 3:**
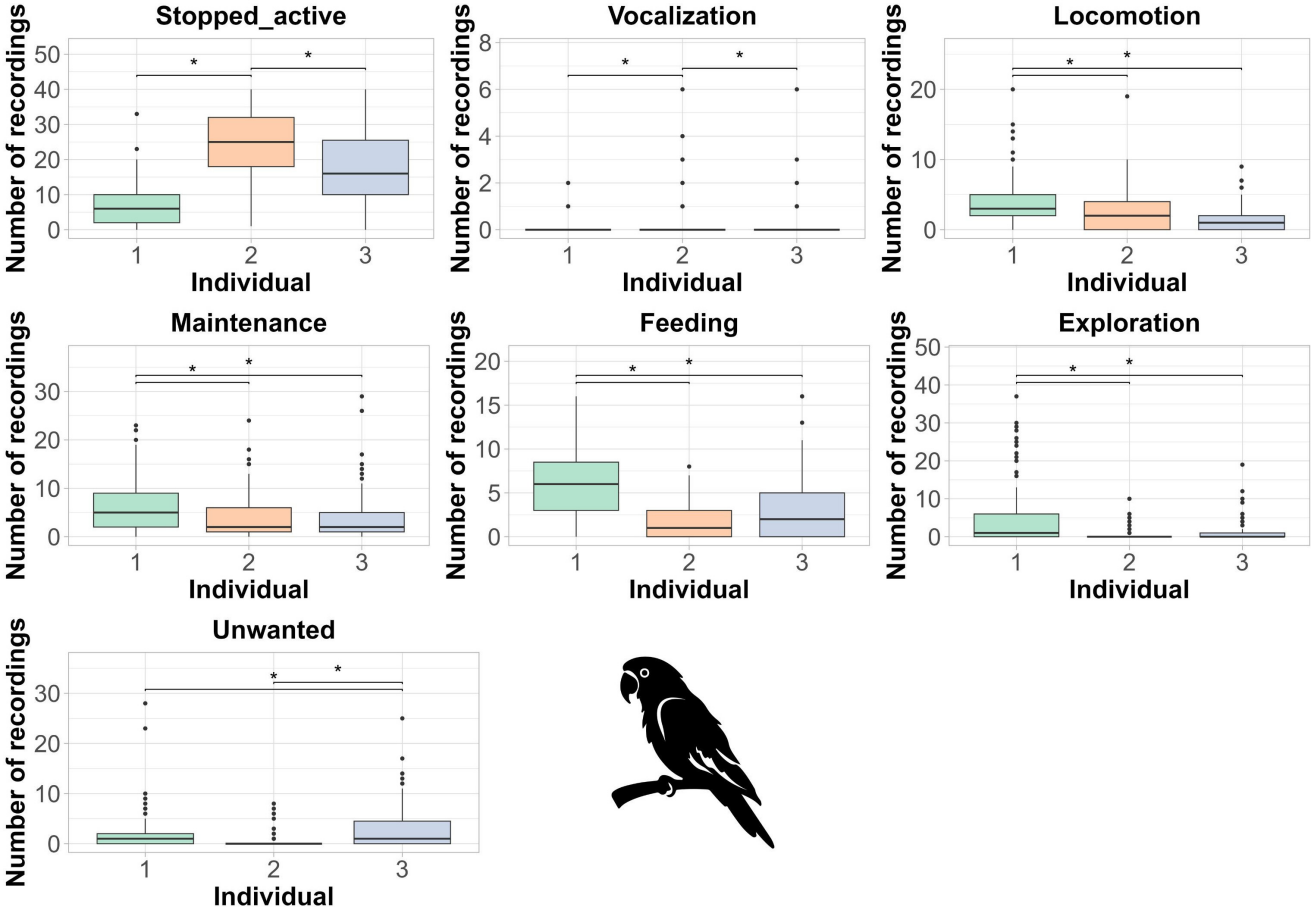
Results of the GLMM for the behaviors exhibited by blue-fronted Amazon parrots (*Amazona aestiva*) in relation to individual birds. * = *p* < 0.05; ** = *p* < 0.01; *** *p* < 0.001. Boxplots: The dark line in the center of the box represents the median, while the lower and upper edges of the box represent the first and third quartiles. The whiskers depict the range of the data, excluding outliers (shown as black dots).

Among individuals, only inactive behavior differed between parrots. Parrot 2 exhibited more stopped active and vocalization behaviors, while parrot 1 exhibited more locomotion, maintenance, feeding and exploration behaviors (Figure 3). Parrot 3 exhibited more unwanted behavior when compared to the others (Figure 3).

**Figure 4:**
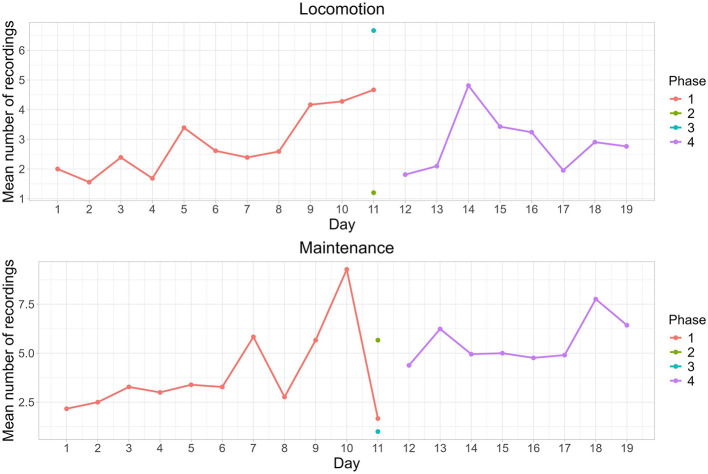
Mean records of locomotion and maintenance behaviors exhibited by the three blue-fronted Amazon parrots (*Amazona aestiva*) during the four phases of the study with pain induction and tramadol treatment (1: Baseline, 2: Induction of osteoarthritis, 3: Post-induction of osteoarthritis with tramadol 1, 4: Post-induction of osteoarthritis with tramadol 2).

Feeding was the only behavior that showed an interaction between the phases and individuals, with individual 2 exhibiting this behavior less frequently than the other individuals in phase 4 (estimate: −0.33, standard error: 0.14, z-value: −2.44, *p* = 0.01). Only the behaviors of locomotion and maintenance were influenced by the day of the study (locomotion: LRT = 7.14, *p* = 0.007; maintenance: LRT = 6.30, *p* = 0.01). Locomotion increased during phase 1, falling to almost zero during pain induction (phase 2). With the administration of tramadol (phase 3), locomotion peaked, then fell and fluctuated on subsequent days (phase 4) (Figure 4). Maintenance behavior was relatively constant at baseline (phase 1) but fluctuated toward the end of this phase. It remained relatively high during phase 2, fell almost to zero in phase 3 and increased in display during phase 4 (Figure 4).

Regarding cage use, overall, parrots used more quadrant A (mean ± SE: 23.83 ± 0.75), followed by quadrant C (mean ± SE: 7.60 ± 0.46), quadrant B (mean ± SE: 4.94 ± 0.45) and quadrant D (mean ± SE: 3.63 ± 0.31). GLMM results showed that birds' use of quadrant A was significantly influenced by phase, individual, and their interaction (Table 2). The individual significantly influenced the use of quadrant B, but the use of quadrant B was not influenced by the phase or the interaction between them (Table 2). The interaction between phase and individual significantly influenced Quadrant C, while the interaction between phase and individual, as well as phase, significantly influenced the use of Quadrant D. Day did not influence the use of any quadrant.

**Table 2:** GLMM results for the cage use by blue-fronted Amazon parrots (*Amazona aestiva*) depending on the study phases (1: Baseline, 2: Induction of osteoarthritis, 3: Post-induction of osteoarthritis with tramadol 1, 4: Post-induction of osteoarthritis with tramadol 2), individuals (1, 2, and 3) and the interaction between these two variables.

Dependent variable	Error distribution	Independent variable	Estimate	z-value	*p*-value
Quadrant A	Gaussian	Phase	−2.82	−2.43	0.02^*^
		Individual	4.13	2.71	0.007^*^
		Phase^*^Individual	1.77	3.29	0.001^*^
Quadrant B	Negative binomial	Phase	0.14	1.00	0.32
		Individual	−2.00	−9.24	< 0.001^*^
		Phase^*^Individual	−0.14	−1.68	0.09
Quadrant C	Negative binomial	Phase	0.19	1.55	0.12
		Individual	0.28	1.83	0.07
		Phase^*^Individual	−0.16	−2.86	0.004^*^
Quadrant D	Negative binomial	Phase	0.34	2.24	0.03^*^
		Individual	0.02	0.11	0.91
		Phase^*^Individual	−0.15	−2.07	0.04^*^

^*^= Statistically significant.

**Figure 5:**
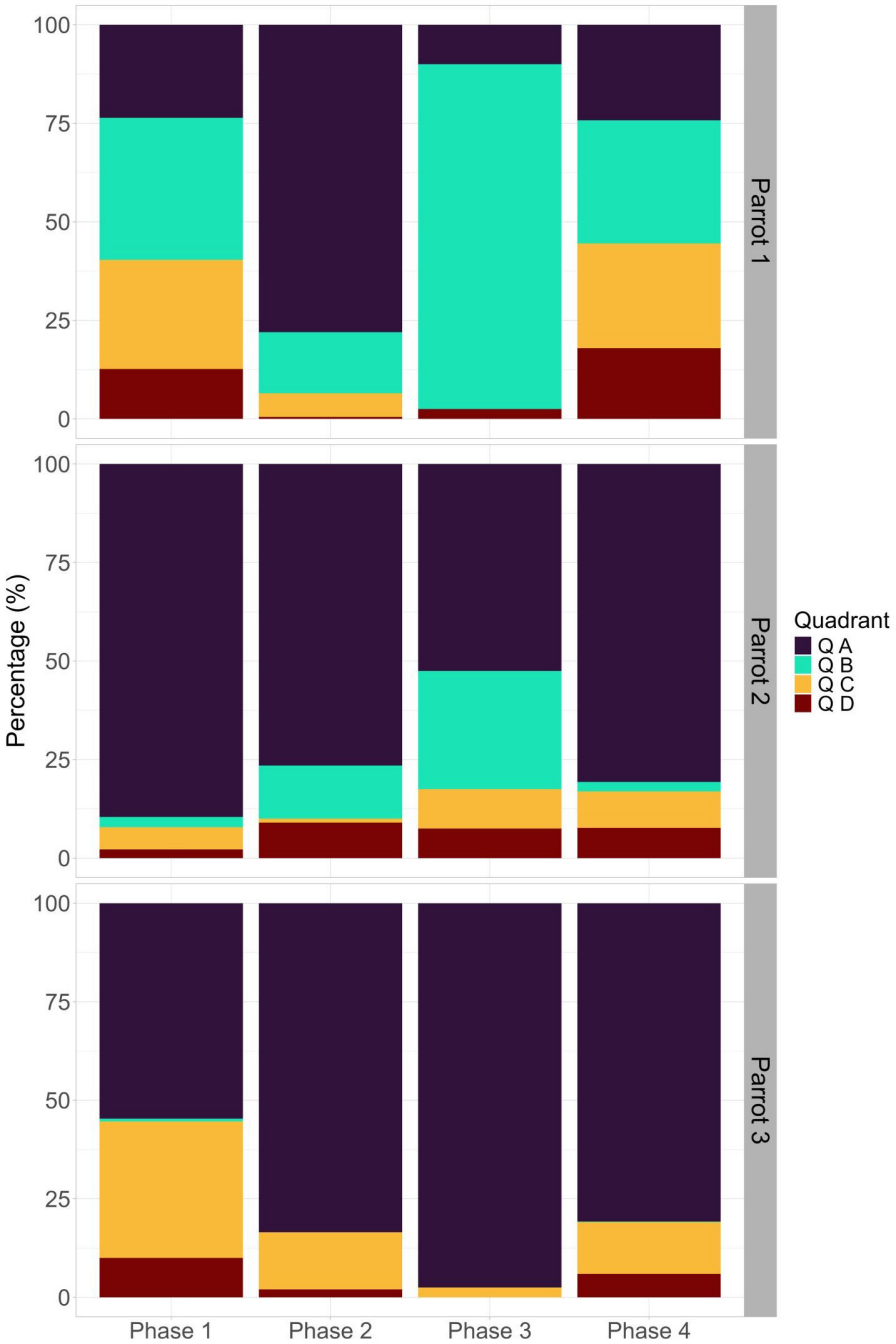
Use of specific quadrants in the cage by the blue-fronted Amazon parrots (*Amazona aestiva*) subjected to experimental osteoarthritis induction and rescue analgesia with tramadol. The study phases are: (1) Baseline; (2) Induction of osteoarthritis; (3) Post-induction of osteoarthritis with tramadol 1; and (4) Post-induction of osteoarthritis with tramadol 2.

Quadrants A and D were used in different ways depending on the phase of the study and the interaction between the phase and the individual. Quadrant A was used more in phase 2 of the experiment, mainly by parrot 2, but parrot 3 also used this quadrant a lot during phase 3 (Figure 5). Quadrant D was used more in phases 4 and 1 of the experiment, especially by parrot 1 (Figure 5). Quadrant C was only influenced by the interaction between the study phase and the individual, being used more by parrot 3 during phase 1 (Figure 5). Finally, quadrant B was used differently by the individuals, with parrot 1 using this quadrant more than the others (Figure 5).

**Figure 6:**
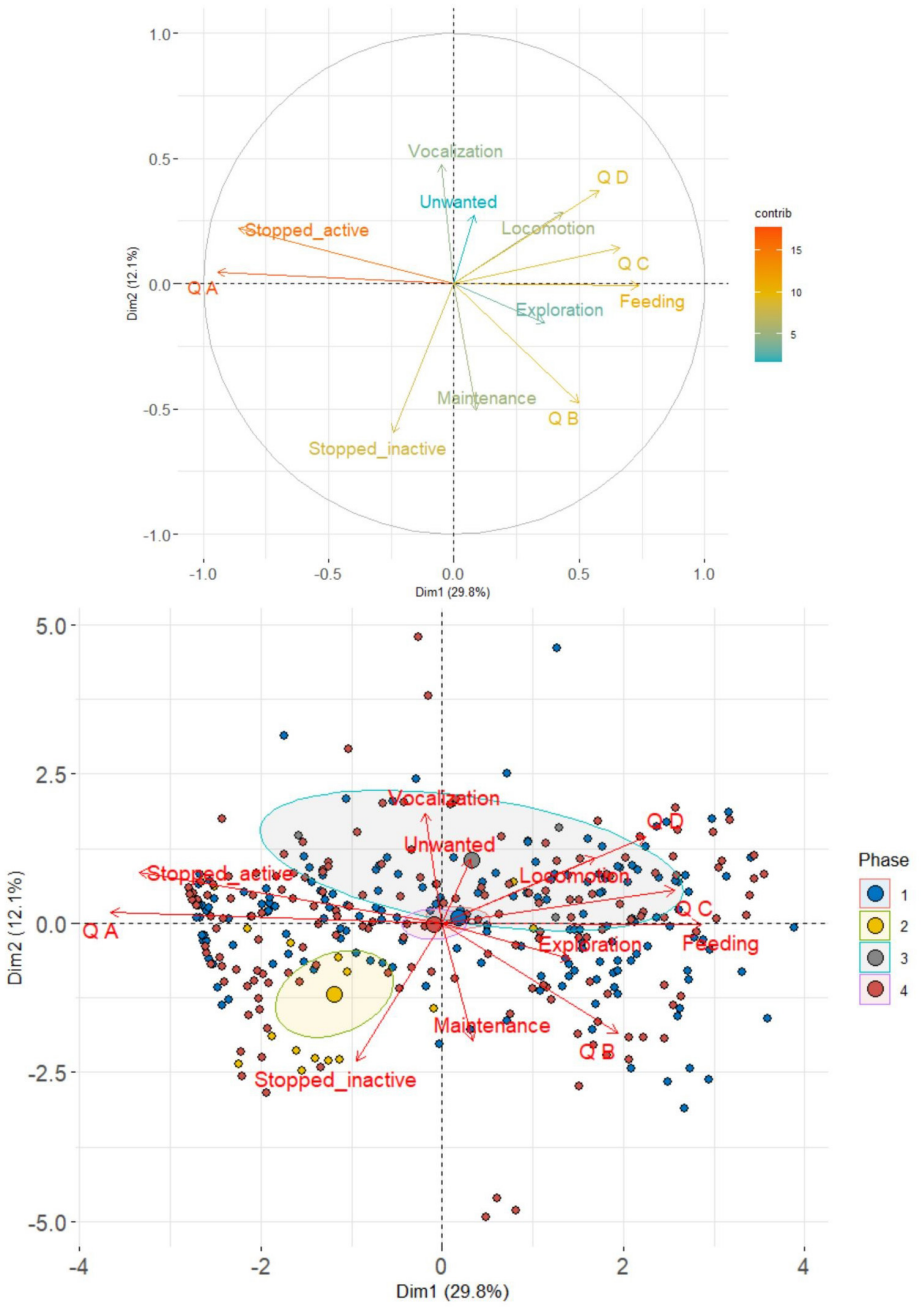
Principal Component Analysis (PCA) results for the behaviors and quadrants used by the three blue-fronted Amazon parrots (*Amazona aestiva*) subjected to experimental osteoarthritis induction and rescue analgesia with tramadol. For PCA results (eigenvalues and variance), see Supplementary material 2.

The first three dimensions of the PCA explained 53.18% of the variance (see Supplementary material 2 for all PCA results). Quadrant A was negatively associated with the behavior Stopped active, especially during the baseline (phase 1) (Figure 6). In contrast, quadrant B was positively related to the behaviors of feeding, exploration, and maintenance (Figure 6). Quadrant D was positively associated with locomotion (Figure 6).

Regarding the use of specific structures and areas in the cage, parrots used more the superior perch (mean ± SE: 22.55 ± 0.73), followed by the inferior perch (mean ± SE: 8.38 ± 0.47), the right posterior corner (mean ± SE: 2.55 ± 0.34), inferior grid (mean ± SE: 1.58 ± 0.27), and the left posterior corner (mean ± SE: 1.10 ± 0.21). All other structures and areas in the cage were used by the parrots less than 1% of the time (anterior grid, drink fountain, fruit, left anterior corner, left grid, posterior grid, ratio feeder, right anterior corner, right grid, superior grid, and treat feeder).

**Figure 7:**
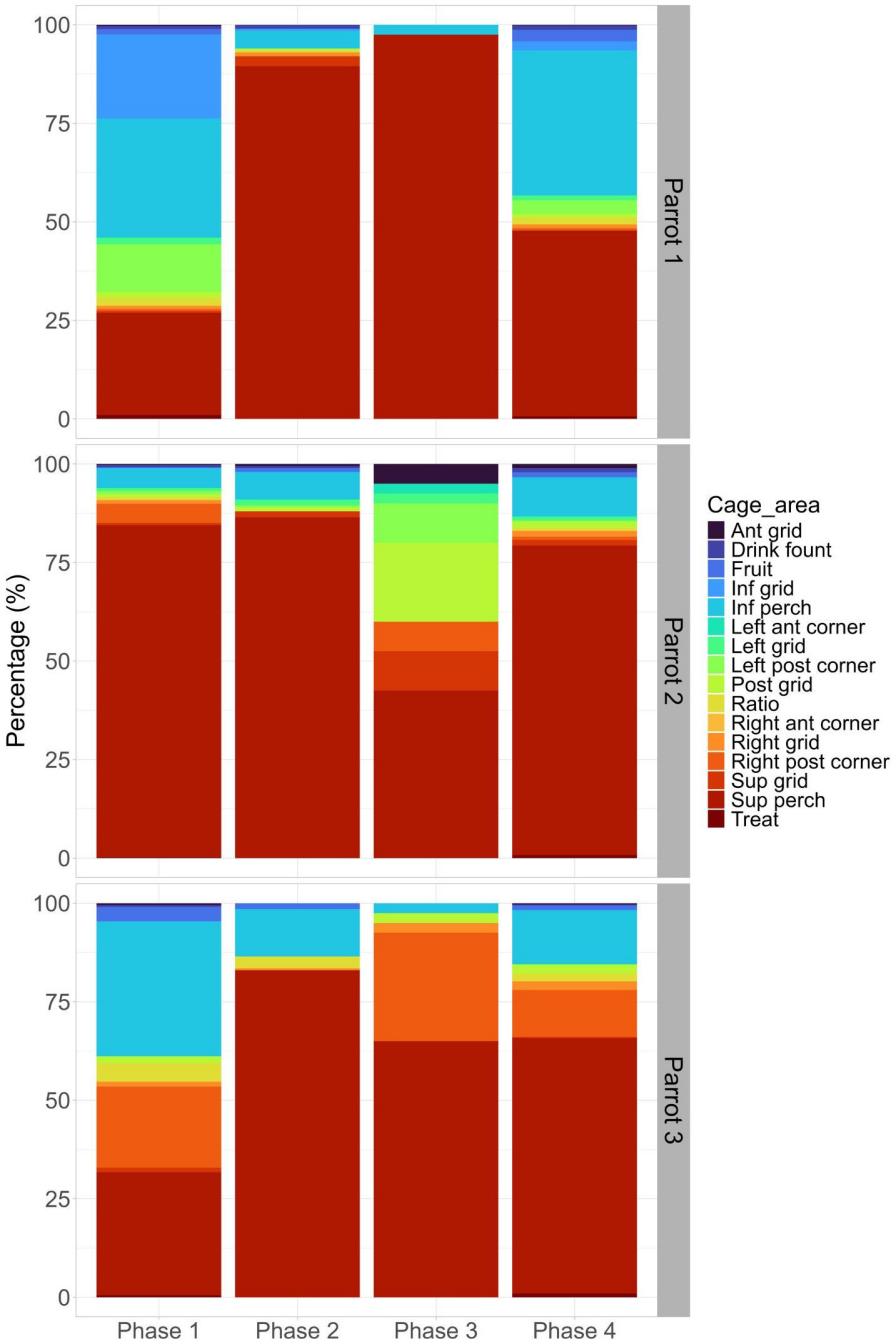
Use of specific structures and areas in the cage by the blue-fronted Amazon parrots (*Amazona aestiva*) subjected to experimental osteoarthritis induction and rescue analgesia with tramadol. The study phases are: (1) Baseline; (2) Induction of osteoarthritis; (3) Post-induction of osteoarthritis with tramadol 1; (4) Post-induction of osteoarthritis with tramadol 2. Ant grid: anterior grid; Drink fount: drink fountain; Fruit: Fruit feeder; Inf grid: Inferior grid; Inf perch: Inferior perch; Left ant corner: Left anterior corner; Left grid: Left grid; Left post corner: Left posterior corner; Post grid: Posterior grid; Ratio: Ratio feeder; Right ant corner: Right anterior corner; Right grid: Right grid; Right post corner: Right posterior corner; Sup grid: Superior grid; Sup perch: Superior perch; Treat: Treat feeder.

GLMM results showed that birds' use of specific cage structures and areas was significantly influenced by phase, individual, and their interaction. The use of the inferior perch (estimate: 0.30, standard error: 0.12, z-value: −2.53, *p* = 0.01) and the fruit feeder (estimate: 0.71, standard error: 0.24, z-value: 2.95, *p* < 0.01) was influenced by the phase, with both areas being used more during phases 1 and 4 (Figure 7). The use of the ratio feeder (estimate: 0.59, standard error: 0.24, z-value: 2.51, *p* = 0.01), fruit feeder (estimate: 0.88, standard error: 0.30, z-value: 2.96, *p* < 0.01), left grid (estimate: −1.46, standard error: 0.36, z-value: −4.12, *p* < 0.01), left posterior corner (estimate: −3.15, standard error: 0.66, z-value: −4.79, *p* < 0.01) and right posterior corner (estimate: 1.89, standard error: 0.33, z-value: 5.74, *p* < 0.01) was influenced by the *individual*, with parrot 1 using more the left grid, parrot 2 using more the left posterior corner, and parrot 3 using more the ratio feeder, the fruit feeder, and the right posterior corner of the cage (Figure 7). Finally, the use of the inferior perch (estimate: −0.19, standard error: 0.05, z-value: −3.44, *p* < 0.01) and fruit feeder (estimate: −0.33, standard error: 0.11, z-value: −3.03, *p* < 0.01) was influenced by the interaction between phase and individual, with the parrots 1 and 3 using more these areas during phases 1 and 4 (Figure 7).

## 4 Discussion

In this study, we hypothesized that the parrots would become less active and would use the cage less during phase 2 of the study due to the induced pain, and that the display levels of most behaviors would return to those observed in phase 1 (baseline), due to the tramadol treatment, which would alleviate the pain in the birds. Our results were consistent with this hypothesis. Although the present study revealed consistent behavioral changes associated with induced osteoarthritis and subsequent tramadol administration, it should be interpreted as an exploratory study. The small sample size (*n* = 3) and the absence of an independent control group limit the strength of causal inferences and the generalizability of the findings. Therefore, the results should be viewed as preliminary evidence contributing to the identification of potential behavioral indicators of pain in *A. aestiva*, rather than as definitive validation of these patterns.

The injection of 8% MUM solution into the left intertarsal joint of parrots induced monoarticular arthritis. This method of inducing arthritic pain simulates the clinical condition of joint uric gout in birds, in which chronic hyperuricemia causes the accumulation of urate crystals in the synovial capsules and tendon sheaths of the joints, thereby compromising weight-bearing in both pelvic limbs and locomotion (39). However, it is important to recognize that this model represents an acute and self-limiting inflammatory condition, which may not fully replicate the complexity of chronic pain observed in naturally occurring clinical cases. This limitation should be considered when extrapolating the present findings to long-term welfare and clinical contexts. Immediately after the procedure, in the interval without interference from analgesic therapy, behavioral changes related to acute arthritic pain were evident, as indicated by a high number of records in the stopped inactive and a low number of records for locomotion and exploration behaviors. The absence of a parallel control or sham-treated group further reinforces this limitation, as it restricts the ability to disentangle the effects of analgesia from time-dependent recovery or behavioral changes associated with the experimental procedures. In addition, although many behavioral observations were obtained, these were derived from only three individuals, raising concerns regarding pseudo-replication and violation of independence assumptions. Consequently, statistical significance should be interpreted with caution, as repeated measures within few individuals may increase the risk of Type I error (40, 41).

Decreased locomotor activity, prolonged resting (inactivity), and support of body weight on the non-arthritic limb have been reported in Hispaniolan parrots, green-cheeked conures, chickens, and domestic pigeons with sodium urate-induced osteoarthritis (14, 15, 42, 43). Reluctance to use the affected limb or to move, as well as protective behaviors associated with injury, are observed in both acute and chronic pain (44, 45).

In untreated osteoarthritis and during the first tramadol administration, a significant difference relative to the baseline period was observed, with reduced feeding. Inappetence is listed among the clinical signs of pain (21). A reduction in food consumption was also observed during sodium urate-induced arthritis in chickens (42).

The high number of registers of the maintenance behavior during osteoarthritis without analgesic therapy may be related to an increase in feather self-care or an attempt to deal with the pain provoked by osteoarthritis. This avian behavior is linked to both acute and chronic pain (46). Excessive preening of the pelvic limb that received an intra-articular injection of 8% MUM solution has been observed in Hispaniolan parrots and green-cheeked conures (14, 15). In domestic chickens with sodium urate-induced arthritis, increased preening and allopreening have been mentioned as an intentional distraction mechanism to attenuate pain and decrease peripheral inflammation (47). Additionally, the ethogram used in this study was based on a relatively short period of preliminary observation, which may have limited the inclusion of less frequent or more subtle behaviors. Although effective for detecting major behavioral changes, further validation with extended observation periods and inter-observer reliability assessments would strengthen its robustness.

A significant increase in the expression of undesired behaviors (stereotypes) was observed during the initial phase of tramadol administration (phase 3), when the parrots were physically restrained for intramuscular injections. Physical restraint is referred to as an acute stressful event in studies that have shown a significant increase in serum corticosterone in Hispaniolan parrots and cockatiels (*Nymphicus hollandicus*) following this procedure (48, 49). Thus, the increase in the exhibition of undesired behaviors was likely linked to the stress resulting from restraint rather than exclusively to the pain caused by osteoarthritis. Handling-related stress should therefore be considered an important confounding factor throughout the experiment. It is possible that other behavioral changes observed across phases, including alterations in locomotion, exploration, and space use, were also influenced by acute stress responses. Disentangling the effects of pain, analgesia, and handling remains challenging and highlights the importance of incorporating control groups and physiological stress indicators (e.g., corticosterone levels) in future studies.

When tramadol (7 mg/kg, IM, BID) was administered 9 and 21 h after inducing osteoarthritis (when the birds were reluctant to move for more than 1 h), there was an improvement in mobility, as indicated by the increased display of locomotion. Progress in exploration, by contrast, remained low even after the final opioid administration relative to baseline. Although not supported by the literature and based on previous observations at the institution where this study was conducted, the 7 mg/kg intramuscular dose of tramadol provided adequate analgesia without adverse effects in *A. aestiva* specimens with induced osteoarthritis. This atypical opioid is recommended for the control of mild to moderate pain, and its analgesic action can be increased with a higher dose. Because not all doses are required across the various situations in avian species, careful adjustments are essential (21). Thus, increased locomotion and minimal exploration may indicate that the tramadol dose effectively alleviated the parrots' pain, but not completely, or that the parrots became afraid to use their entire cage due to stress induced by capture. Although the observed behavioral improvements suggest a potential analgesic effect of tramadol, this interpretation should be made with caution. The lack of a control group and the absence of comparison with alternative analgesic protocols prevent definitive conclusions regarding its efficacy. In addition, potential adverse effects of tramadol were monitored behaviourally throughout the study period, including signs such as sedation, abnormal posture, reduced responsiveness, or gastrointestinal alterations. No overt adverse effects were observed under the conditions of this study. However, given the small sample size and the absence of physiological or biochemical monitoring, subtle or transient effects may have gone undetected. Furthermore, the dose used in this study (7 mg/kg IM) differs from those reported in the literature for other avian species, which vary considerably depending on species, route, and study design. For instance, in Hispaniolan Amazon parrots (*Amazona ventralis*) and Muscovy ducks (*Cairina moschata*), oral tramadol at 30 mg/kg produced plasma concentrations associated with analgesia (50, 51). However, in American kestrels (*Falco sparverius*), oral tramadol at 5 mg/kg provided antinociceptive effects (52), and in bald eagles (*Haliaeetus leucocephalus*), an oral dose of 11 mg/kg has been reported (53). Moreover, most pharmacokinetic and pharmacodynamic studies in psittacine birds have focused on oral administration (54, 55), whereas the present study used the intramuscular route. Given the well-documented interspecific variability in tramadol metabolism, absorption, and bioavailability across avian species, including marked differences even among psittacines (55), direct extrapolation of dosing regimens is limited (8). Future studies exploring dose-response relationships, species-specific pharmacokinetics, and comparative efficacy of different routes of administration in *A. aestiva* are necessary to better support its clinical application.

Regarding cage use, the parrots in this study occupied the perches, especially the superior one, more than any other specific area in the cage. Staying on the highest perch was intensified by a substantial expression of inactivity due to untreated arthritic pain. Perching is a strongly ingrained anti-predation behavior in birds (56). Occupying the highest perch is considered an advantage, as in the wild, being closer to the ground means greater exposure to danger (57). The significant occupation of quadrant A during phase 2 is mainly correlated with the use of the superior perch, the structure with the highest number of accesses. The notable use of quadrant C in the baseline period (phase 1) is mainly attributed to the use of the inferior perch (the second most used specific site). By inducing osteoarthritis, the pain caused the birds to become more inactive and to use fewer areas and structures in the cage. After tramadol administration, more activity and use of space were recorded, supporting the interpretation that tramadol may have contributed to behavioral recovery in the parrots.

Some behaviors were influenced by the individual, as was the use of the cage and its structures. Individuals 2 and 3 were more reactive in the experiment, exhibiting fewer stopped-active and unwanted behaviors and more locomotion, feeding, and exploration behaviors. Individual 1 did not change his behavior significantly and continued to use the cage and its structures more frequently than the other individuals. These results demonstrate how the personality of each parrot can influence its responses to the stress induced by the experiment and the handling during phases 2, 3, and 4. Different reactions to handling have already been recorded for both the blue-fronted Amazon parrot and other psittacine species, such as the vinaceous-breasted Amazon parrot (*A. vinacea*) and the red-browed Amazon parrot (*A. rhodocorytha*); these differences have been linked to variations in personality (58). Thus, future studies could, in addition to increasing the sample size, evaluate personality-related aspects. An interesting observation from the study is that, during the osteoarthritis induction phase (phase 2), all three parrots used the upper area of the cage, either on the perch or on the grid. Even without seeing each other, the birds remained in the places closest to one another in each cage. This behavior could be an indication that, in a situation of pain, the birds seek other individuals to be more grouped (in the present experiment, using vocalizations to get closer to each other), which could increase the individuals' sense of security (dilution effect) (59), which might increase the speed of healing since birds would avoid spending energy on vigilance. Future studies could investigate this hypothesis.

To date, this is the first study to experimentally induce osteoarthritis in blue-fronted Amazon parrots by injecting them with an 8% MUM solution intra-articularly, using tramadol as a rescue analgesic, and to evaluate changes in their behavioral patterns in a laboratory environment. By comparing analgesic protocols, increasing the sample size, and employing a crossover design, future studies can yield more accurate results, thereby enhancing knowledge about the use of behavioral methods to assess pain in psittacine birds.

In conclusion, the purpose of the study was to identify changes in the behavioral patterns of blue-fronted Amazon parrots subjected to experimental induction of osteoarthritis and rescue analgesia with tramadol in a laboratory environment. In conclusion, it was found that injecting 8% MUM solution into the left intertarsal joint induced monoarticular osteoarthritis in the parrots, simulating the condition of joint uric gout. In addition, the changes observed in some behavioral patterns, such as a decrease in locomotion and exploration, and an increase in stopped inactive behaviors, as well as in the use of the cage (fewer areas used during acute pain), were consistent with the manifestation of unilateral arthritic pain and its apparent attenuation following tramadol administration. In this way, the species-specific ethogram for arthritic pain developed in this study may inform future antialgic evaluations of tramadol and other drugs, enabling the construction of a pain scale for *A. aestiva*. Further studies with the blue-fronted Amazon parrot and other psittacine species are essential to improve recognition of pain-related behaviors in human care.

This study has some limitations that should be acknowledged. First, the sample size was relatively small (*n* = 3), reflecting the ethical and logistical constraints inherent in experimental studies involving companion avian species. Although this is common in controlled pharmacological trials with birds, the limited number of individuals reduces statistical power and restricts the generalizability of the findings. Moreover, although many behavioral observations were collected, these were derived from the same individuals, which raises concerns regarding pseudo-replication and potential violation of independence assumptions, requiring cautious interpretation of statistical significance. In addition, the absence of a parallel untreated or sham control group limits the ability to fully distinguish drug-related effects from time-dependent recovery, handling-related stress, or individual variability. Handling procedures, particularly physical restraint for drug administration, likely acted as a confounding factor influencing behavioral responses across phases. Responses to analgesic protocols may also differ across species, life stages, and clinical conditions, and interspecific variability in tramadol metabolism may influence pharmacological outcomes. Therefore, extrapolation beyond the studied population should be made with caution. Taken together, these constraints indicate that the present findings should be interpreted as preliminary but meaningful contributions to the understanding of pain-related behavior in *A. aestiva*. Future studies with larger sample sizes, inclusion of control groups, and integration of behavioral, physiological, and pharmacological approaches will be essential to strengthen causal inference and support clinical application.

## Data Availability

The datasets presented in this study can be found in online repositories. The names of the repository/repositories and accession number(s) can be found below: The datasets generated and analyzed for this study can be found in the Mendeley Repository Data (https://doi.org/10.17632/h9246zcsmt.1).
