# Supplementary material for: Behavioral assessment of blue-fronted Amazon parrots (Amazona aestiva Linnaeus, 1758) subjected to experimentally induced osteoarthritis and rescue analgesia with tramadol in a laboratory environment
Source: Front Vet Sci. 2026 May 14;13:1802230. doi: 10.3389/fvets.2026.1802230 (PMC13215933; doi:10.3389/fvets.2026.1802230)
Supplement: Supplementary file 2 [file Table_2.docx]

Supplementary Material

# Supplementary Data

Supplementary Material should be uploaded separately on submission. Please include any supplementary data, figures and/or tables.

Supplementary material is not typeset so please ensure that all information is clearly presented, the appropriate caption is included in the file and not in the manuscript, and that the style conforms to the rest of the article.

# Supplementary Figure

Training was conducted using nine cadavers of blue-fronted Amazon parrots (*Amazona aestiva*) to induce osteoarthritis through the injection of a monosodium urate monohydrate (MUM) solution. During the training, the MUM solution was replaced with 1% methylene blue dye (PERFYL TECH®) in a 1 mL syringe (SR®) coupled to a 23 G needle (SR®); a volume of 0.1 mL was injected into both intertarsal joints of the nine parrot corpses.

## Supplementary Figures


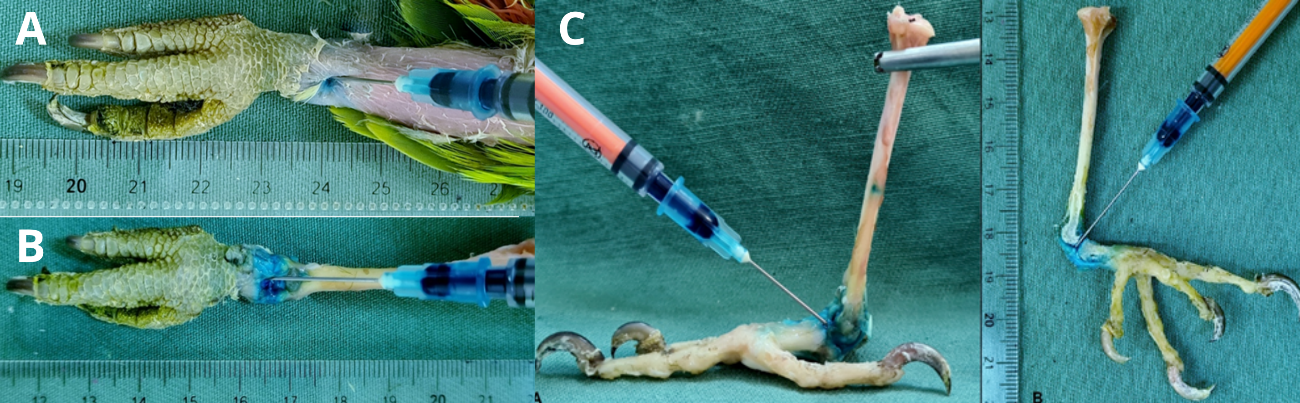


**Supplementary Figure 1.** A) Dorsal view of the left intertarsal joint of a blue-fronted Amazon parrot (*Amazona aestiva*) after removal of the tibiotarsal feathers and injection of 10% methylene blue solution. B) Dorsal view of the 10% methylene blue solution injection site in the left intertarsal joint of the parrot after removal of the tibiotarsal soft tissues. C) Lateral view of the needle at the site of injection of a 10% methylene blue solution into the left intertarsal joint of a blue-fronted Amazon parrot, following removal of the soft tissues of the tibiotarsus, tarsometatarsus, and phalanges (left), and medial view of the same region (right).
